# Disease activity and disease-related factors are drivers of patient global assessment in rheumatoid arthritis: a real-life cross-sectional study

**DOI:** 10.1007/s00296-023-05383-6

**Published:** 2023-07-16

**Authors:** Ilaria Suardi, Cristina Posio, Ester Luconi, Patrizia Boracchi, Roberto Caporali, Francesca Ingegnoli

**Affiliations:** 1 Rheumatology Clinic, Department of Clinical Sciences and Community Health, ASST Gaetano Pini-CTO, Università degli Studi di Milano, Milan, Italy; 2 Università degli Studi di Milano, Department of Biomedical and Clinical Sciences “L. Sacco”, Milan, Italy

**Keywords:** Rheumatoid arthritis, Disease activity, Patient-physician discordance, Patient Global Assessment, Remission

## Abstract

Despite that the Patient Global Assessment (PGA) is widely used for measuring Rheumatoid Arthritis (RA) disease activity to define the remission state of the disease, the primary contributors influencing patients’ ratings are still debated. This study aims to determine which clinical, sociodemographic and lifestyle-related contextual factors might be key drivers of PGA in RA. This single-center cross-sectional study recruited 393 consecutive adult RA patients. Median age 60 years, females 306 (77.9%). Data related to disease activity were assessed by using Simplified Disease Activity Index (SDAI), severity by Health Assessment Questionnaire (HAQ), and impact by RA Impact of Disease (RAID). Sociodemographic/lifestyle features were collected. Disease remission was calculated using Boolean-based criteria 1.0 and 2.0. Quantile regression models were used for univariate and multivariate analysis. The remission rate progressively increased from 15% by using SDAI with a Boolean 1.0-based definition to 43.5% using a Boolean 2.0-based remission. Among factors related to disease activity, the use of low-dose corticosteroids, the RAID items pain and sleep difficulties were predictive for worse PGA scores (*p* = 0.01). Among factors related to disease severity HAQ score and RAID total were independent factors associated with higher median PGA (*p* = 0.02 and *p* < 0.001). RAID’s physical well-being was related to PGA scores (*p* = 0.01). An increasing trend in PGA was observed in longstanding diseases (> 15 years). Our results confirmed that there is no unambiguous interpretation of the PGA score. It is a measure related to some disease activity parameters, but it is also influenced by contextual factors related to disease severity and impact. These data highlighted that PGA should have a broad interpretation, thus supporting the proposal of a dual targets (biological and impact) approach to obtain a more accurate estimate of disease activity.

## Introduction

Rheumatoid arthritis (RA) is a chronic, systemic, inflammatory, immune-mediated disease characterized by sustained synovitis in multiple joints. In addition, extra-articular complications may also occur throughout the disease course [1]. Nowadays, treat-2-target (T2T) principles represent the best approach for achieving a clinical remission state, *i.e.* an improvement in clinical outcomes, quality of life and the prevention of structural damage [2, 3]. The remission concept is defined as no or minimal disease activity as reflected by several composite measures such as the Disease Activity Score in 28 joints (DAS28), the Clinical Disease Activity Index (CDAI), the Simplified Disease Activity Index (SDAI), and American College of Rheumatology (ACR)/European League Against Rheumatism (EULAR) Boolean remission criteria [4]. These activity indexes, aside from the set of pivotal clinical variables such as tender and swollen joint count (TJC, SJC), and acute phase reactants (C reactive protein -CPR- or erythrocyte sedimentation rate -ESR-), include the patient’s perspective directly captured through a patient-reported outcome (PRO) measure called the “patient global assessment” (PGA) of disease activity [5, 6].

Some critical aspects of this latter parameter (e.g., different formulations, difficulties in interpreting and rating the score) have led to an ongoing debate as to whether PGA could be considered a reliable and appropriate tool to measure disease activity consequently guiding immunosuppressive therapy. In addition, albeit PGA should provide valuable insight into the RA condition regarding aspects reliant on disease activity such as pain, functional ability, stiffness, and joint swelling, it can often capture factors related to RA severity and impact due to the complexity of the disease [7]. Currently, the available remission criteria suggest that the PGA question should be formulated as follows: “Considering all the ways your arthritis has affected you, how do you feel your arthritis is today?”. It can be applied using a numeric rating scale (NRS), a verbally administered NRS, or a visual analogue scale (VAS) ranging from 0 to 100 mm or 0–10 cm, where higher scores represent a higher level of disease activity or worse global health [8].

Similarly, also the Physician Global Assessments of disease activity (PhGA), which is calculated according to the same criteria (0–100 mm or 0–10 cm), plays an important role in evaluating RA progression since it is included in both CDAI and SDAI indices. Interestingly, even though both PGA and PhGA are conceived to reflect disease activity, it has been shown that in daily care practice, roughly one-third of RA patients report higher scores compared to those indicated by their providers [9]. Specifically, previous studies have observed that, while physicians tend to consider objective aspects such as physical evaluation findings, imaging and laboratory results and use of corticosteroids, patients’ perspective seems to be primarily influenced by their general well-being. This encompasses an elaborate consideration of diverse factors including pain, fatigue, functional disability (evaluated by HAQ — Health Assessment Questionnaire), presence of comorbidities, fibromyalgia (according to ACR 2016 criteria) [10], and psychological distress (mainly depression and anxiety) [11–13]. Pain in particular (either due to RA or other medical issues) has proved to account for up to 75% of the PGA result regardless of inflammatory status [14, 15]. Such discrepancies can negatively affect the achievement of the remission state, mostly when there are no signs of visible inflammation and PGA is the only abnormal parameter (> 1 as indicated by the Boolean definition) [16, 17]. As a consequence, patients would be labelled as “near remission” instead of “in remission”, leading to non-appropriate management of immunosuppressive treatment that they would not benefit from instead of adjuvant interventions [18, 19].

Since PGA is often related to difficulties in interpretation, thus sometimes becoming the only parameter limiting the achievement of remission; this study aims to explore which aspects of disease activity describe PGA and whether contextual factors such as disease severity, burden, and sociodemographic and lifestyle variables might influence the PGA scores.

## Materials and methods

### Study design

This observational cross-sectional study recruited consecutive patients diagnosed with RA from the ASST Gaetano Pini-CTO, Milan. Inclusion criteria were as follows: (1) fulfilment of the 1987 American College of Rheumatology (ACR) [20] and/or 2010 ACR/European League Against Rheumatism classification criteria for RA [21] referring to our in- and out-patient rheumatology clinic; (2) age ≥ 18 years; (3) disease duration ≥ 3 months. Exclusion criteria were: (1) inability to understand the privacy policy and give the informed consent; (2) presence of overlap syndromes. The local ethical committee approved the study (Ethics Committee 138_1999), and all participants gave written informed consent.

**Fig. 1:**
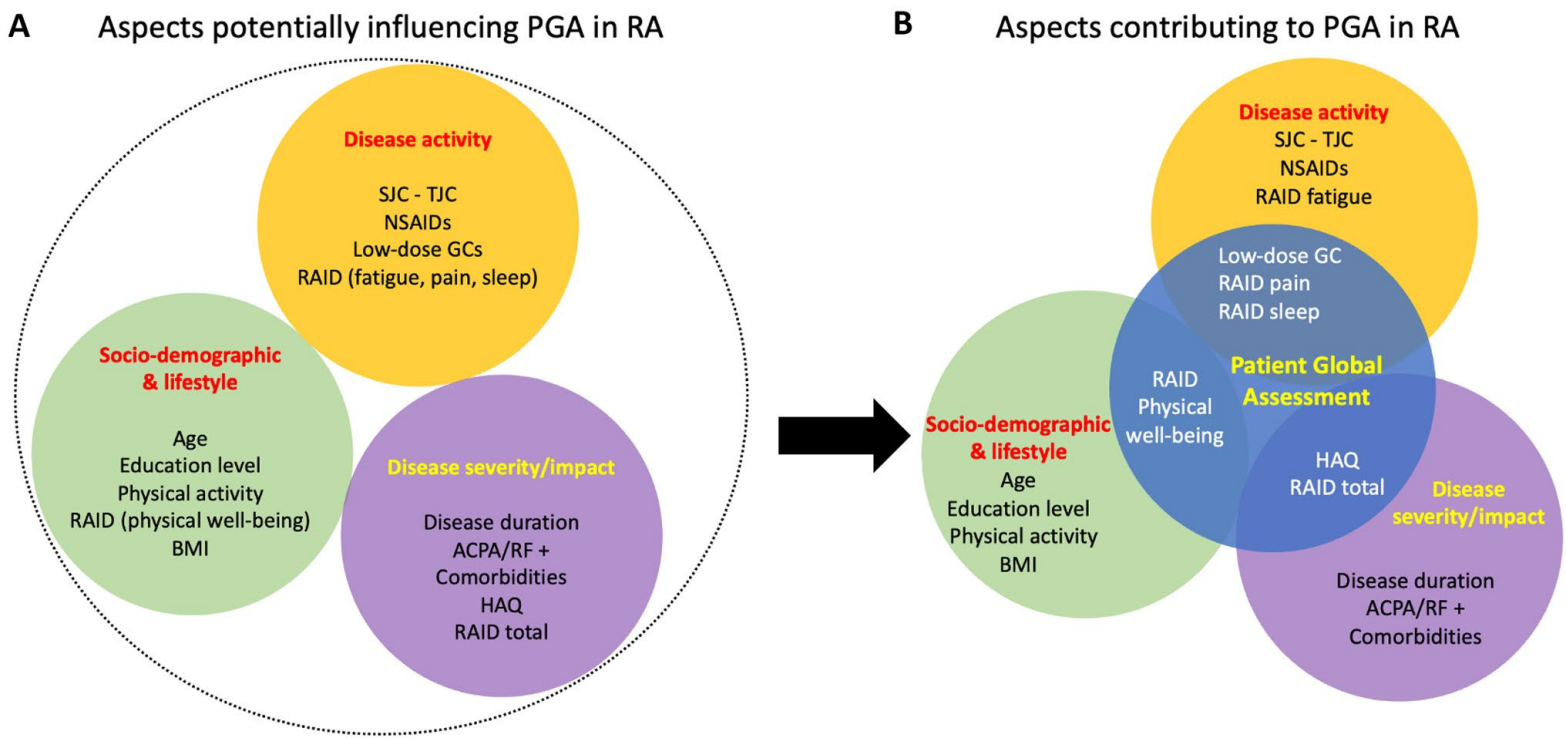
Aspects related and unrelated to disease activity included in this study as potentially influencing the PGA rating. The study hypothesis (**A**) and summary of results (**B**). *ACPA* anti-citrullinated protein antibody, *BMI* body mass index, *HAQ* health assessment questionnaire, low-dose GC (glucocorticoids): ≤ 7.5 mg/day prednisone or equivalent, *NSAIDs* non**-**steroidal anti-inflammatory drugs, *PGA* patient global assessment; *RAID* rheumatoid arthritis impact of disease score, *RF* rheumatoid factor *SJC*: swollen joint count, *TJC* tender joint count

In this work, we aimed to identify the potential aspects that could influence the PGA score in RA patients. Thus, we tested whether variables related and unrelated to disease activity could drive PGA scoring. In particular, three macro-areas were considered: (1) disease severity/impact; (2) disease activity; and (3) socio-demographic and lifestyle variables (Fig. 1A).

### Data collection

During the rheumatologic visit, we collected data related to the above-mentioned macro-areas, as well as laboratory variables and PROs.

Among socio-demographic data, we recorded age at visit, gender, and educational level. Lifestyle included body mass index (BMI), and weekly hours of physical activity, assessed through a self-administered questionnaire. Clinical parameters included: disease duration (years), TJC28, SJC28, and comorbidities such as gastroesophageal reflux disease (GERD), Inflammatory Bowel Diseases (IBD), Gastritis, Cardiovascular Diseases (CD), arterial hypertension, esophagitis, Diabetes Mellitus (DM), fibromyalgia and depression. Laboratory parameters considered the presence or absence of rheumatoid factor (RF) and anti-cyclic citrullinated peptide antibody (ACPA), and CRP (mg/dL) level. Moreover, ongoing therapies and drug history were recorded.

The following PROs were also collected: (1) the PGA was assessed using the formulation recommended by ACR/EULAR: “considering all the ways your arthritis has affected you, how do you feel your arthritis is today?”, ranging from 0 (best disease control) to 10 (worst disease control) on a visual analogue scale; (2) the Rheumatoid Arthritis Impact of Disease (RAID), a multidimensional scale developed by EULAR which assesses seven domains: pain, functional disability, fatigue, sleep, physical/emotional well-being, and coping; each of them is scored on a 10-item numerical rating scale, with 0 indicating low disease activity and 10 high disease activity [22]; (3) the Health Assessment Questionnaire (HAQ), a common tool for the assessment of functional ability in RA; it consists of 20 items grouped into eight categories concerning different functional areas. For each item, the patient is asked to score the difficulty on a six-point scale; higher scores are associated with substantial difficulties in daily life [23].

PhGA was assessed through a visual analogue scale ranging from 0 (best disease control) to 10 (worst disease control) [24].

Disease activity was calculated using both Boolean-based criteria and SDAI. ACR/EULAR Boolean remission definition comprises SJC, TJC, CRP level (mg/dL) and PGA; remission is obtained when all variables have a rating ≤ 1 (Boolean 1.0) [24]. PGA-near-remission state is used to describe patients in which PGA is the only factor with a score > 1 that limits the reaching of remission [16, 17]. Therefore Boolean 2.0 remission, recently endorsed by ACR/EULAR revision criteria, provides a higher PGA threshold of 2 cm on a 0–10 cm scale [4, 25], showing a better concordance with SDAI. This latter index, in addition to the above-mentioned components, includes also PhGA and in this case remission is defined by a sum score ≤ 3.3 [26].

### Statistical methods

For categorical variables number of patients and percentages were reported for each category. The distribution of the variables measured on numerical scale were synthesized reporting the median and the IQR (interquartile range).

To evaluate the association between PGA and the considered variables quantile regression models were used in both univariate and multivariate analysis [27]. For each model, the median of the PGA was considered as the response variable, while other variables were considered as covariates. The inference on regression coefficients (hypothesis tests and confidence intervals) were based on the bootstrapping technique (10,000 bootstrap samples). Categorical variables were included by a dummy coding. One of the category was defined as reference and model results are reported as the difference between the median PGA of each category and the median PGA of the reference one together with 95% confidence intervals (95% C.I.). Numerical variables were included in their original measurement scale. The existence of a non-linear relationship was assessed by the use of three knots restricted cubic spline; if the non-linear spline term was not statistically significant, only a linear relationship was considered, and results are reported as the variation of PGA median for each unit increase of the numerical covariate together with 95% confidence intervals (95% C.I.). In the presence of non-linear relationship regression coefficients are not directly interpretable thus model results are reported by a graphical representation of the estimated relationship between PGA median and covariate values.

When there was a strongly asymmetrical distribution of covariate values, to avoid unreliable results, the covariate was transformed into categorical one by suitable cut-offs.

To avoid regression models including a large number of variables and to facilitate results interpretation, separate analyses were performed after subdividing the variables according to the three macro-areas. Specifically, among socio/demographic and lifestyle variables we considered: age at visit (18–59 years *vs* ≥ 60), education level (primary/secondary *vs* academic education), physical activity (sedentary *vs* physical activity 1–2 times/week *vs* physical activity > 2 times/week), BMI and RAID physical well-being (0–10).

For the “disease severity/impact” section we considered HAQ (0–3), disease duration (with a spline terms), ACPA and/or RF positivity (yes/no), comorbidities (none, 1 comorbidity and > 1 comorbidity) and RAID total score (0–10).

Finally, as for disease activity, we investigated: TJC (0–1 vs > 1), SJC (0–1 *vs* > 1*),* use of non-steroidal anti-inflammatory drugs (NSAIDs) and corticosteroids *(yes/no),* RAID domains “pain”, “fatigue” and “sleep” (0–10).

The final multivariate reduced model was obtained by a “backward” selection procedure Specifically, at each step of the backward procedure, the variables with *p* >  = 0.1 were removed.

The significant variables for the multivariate models were inserted into a new model of quantile regression and the estimation of the goodness of fit with those variables was assessed using Koenker and Machado’s pseudo-R2 [28].

Furthermore, we explored whether some of the patients met the EULAR criteria for the definition of “difficult to treat” [29]: (1) failure of ≥ 2 biological disease-modifying antirheumatic drugs (DMARDs)/targeted synthetic DMARDs (with different mechanisms of action) after failing conventional synthetic DMARD therapy (unless contraindicated); (2) presence of at least one of the following: at least moderate disease activity; signs and/or symptoms suggestive of active disease; inability to taper glucocorticoid treatment; rapid radiographic progression; RA symptoms that are causing a reduction in quality of life; (3) the management of signs and/or symptoms is perceived as problematic by the rheumatologist and/or the patient.

The agreement between PGA and PhGA was assessed through Lin's concordance correlation coefficient [30].

## Results

### Study population

The study included 393 patients with a diagnosis of RA who regularly attended our in- or outpatient rheumatologic clinic. Descriptive statistics and/or frequency distributions of demographic characteristics, clinical and serological features, comorbidities, and ongoing treatments of the RA patients included in the current study are reported in Table 1.

**Table 1:** Demographics and disease characteristics of rheumatoid arthritis patients

	Rheumatoid arthritis cohort (*n* = 393)
Age, years (median, IQR)	59 (48–70)
Female *n* (%)	306 (77.9%)
Educational level	
Primary *n* (%)	56 (14.2%)
Lower secondary *n* (%)	93 (23.7%)
Upper secondary *n* (%)	141 (35.9%)
Academic n (%)	91 (23.1%)
BMI (median, IQR)	23 (21–26)
Physical activity	
Sedentary *n* (%)	119 (73%)
Disease duration [years, median (IQR)]	8 (13–21)
FR, *n* (%)	212 (53.9%)
ACPA, *n* (%)	199 (50.6%)
TJC > 1, *n* (%)	106 (26,97%)
SJC > 1, *n* (%)	74 (18.82%)
Comorbidities	
Cardiovascular disease *n* (%)	23 (5.8%)
Diabetes n (%)	24 (6.1%)
Arterial hypertension *n* (%)	138 (35.1%)
Lung disease *n* (%)	15 (3.8%)
GERD *n* (%)	78 (19.8%)
Fibromyalgia *n* (%)	41 (10.4%)
Depressive symptoms *n* (%)	19 (5.3%)
Therapy	
Low-dose GC *n* (%)	172 (43.8%)
NSAIDs *n* (%)	65 (16.6%)
Only csDMARDS *n* (%)	165 (42%)
Only ts/bDMARDs *n* (%)	90 (22.9%)
csDMARDS + ts/bDMARDs *n* (%)	96 (24.4%)
No therapy	42 (10.7%)

*ACPA* anti-citrullinated protein antibody, *bDMARD* biological DMARD, *BMI* body mass index, *csDMARD* conventional synthetic *DMARD*, *DMARD* disease-modifying anti-rheumatic drug, *GERD* gastroesophageal reflux disease, *low-dose GC (glucocorticoids)* ≤ 7.5 mg/day prednisone or equivalent, *NSAIDs* non**-**steroidal anti-inflammatory drugs, *RF* rheumatoid factor, *SJC* swollen joint count, *TJC* tender joint count, *tsDMARD* targeted synthetic DMARD

Briefly, the median age (IQR) at the visit was 60 (49–70) years, females were 306 (77.9%), and most of the participants [232, (59%)] finished high school and/or university. The median BMI was 23.73 kg/m2. The median (IQR) disease duration was 8 (13–21) years, with RF positivity being 212 (53.9%) and ACPA positivity of 199 (50.6%).

Major comorbidities included arterial hypertension [138 (35.1%)], GERD [78 (19.8%)] and gastritis [36 (9.16%)]. Only 26 patients (6.61%) met the D2T criteria of whom descriptive statistics are reported in Table 2.

**Table 2:** Difficult to treat cohort: demographic details, clinical features, and PROs

	Difficult-to-treat (*n* = 26)
Demographics	
Age, years (median, Q1–Q3)	60 (46–68)
Female *n* (%)	25 (96.2%)
BMI (median, Q1–Q3)	23 (21–26)
Educational level	
Primary *n* (%)	3 (11.5%)
Lower secondary *n* (%)	7 (26.9%)
Upper secondary *n* (%)	10 (38.5%)
Academic *n* (%)	6 (23.07%)
Disease	
Disease duration [years, median (IQR)]	19.6 (15.2–31.9)
FR, *n* (%)	20 (76.9%)
ACPA, *n* (%)	17 (65.38%)
TJC > 1, *n* (%)	21 (80.76%)
SJC > 1, *n* (%)	17 (65.38%)
Comorbidities	
Cardiovascular disease *n* (%)	23 (5.8%)
Diabetes *n* (%)	2 (7.7%)
Arterial Hypertension *n* (%)	10 (38.5%)
Lung disease *n* (%)	0 (0%)
GERD *n* (%)	7 (26.9%)
Fibromyalgia *n* (%)	7 (30.4%)
Depressive symptoms *n* (%)	1 (4.3%)
Therapy	
Low-dose GC *n* (%)	18 (69.2%)
NSAIDs *n* (%)	14 (53.8%)
Patient reported outcome	
PGA (median, IQR)	5 (3.5–5)
HAQ (median, IQR)	1 (0.62–1.06)
RAID (median, IQR)	6.07 (4.35–6.55)

*ACPA* anti-citrullinated protein antibody, *BMI* body mass index, *GERD* gastroesophageal reflux disease, *HAQ* Health Assessment Questionnaire, *low-dose GC (glucocorticoids)* ≤ 7.5 mg/day prednisone or equivalent, *NSAIDs* non-steroidal anti-inflammatory drugs, *PGA* Patient Global Assessment, *RAID* Rheumatoid Arthritis Impact of Disease score, *RF* rheumatoid factor, *SJC* swollen joint count, *TJC* tender joint count

### PGA and rate of remission

The median of PGA reported in the whole cohort was 3.13 (IQR 2–4.5). According to ACR/EULAR remission criteria, the overall remission rate in this cohort was 28% (108 patients with SDAI < 3.3), while the remission rate assessed through Boolean 1.0-based definition, was only 15%. However, the remission rate increased up to 26.2% (*n* = 102) following Boolean 2.0 based remission (TJC, SJC and CRP (mg/dL) ≤ 1 with PGA score < 2) and up to 43.5% (*n* = 171) considering also near remission state (TJC, SJC and CRP (mg/dL) ≤ 1 with PGA score > 1).

Consistently with these results, Lin's concordance correlation coefficient between PGA and PhGA was only 0.4. Remission rates are shown briefly in Table 3.

**Table 3:** Remission rate according Boolean-based criteria and index-based criteria (SDAI)

Simplified disease activity index	
Remission = 0.0–3.3 (*n*, %)	108 (28%)
Low activity = 3.4–11.0 (*n*, %)	167 (43%)
Moderate activity = 11.1–26.0 (*n*, %)	86 (22%)
High activity = 26.1–86.0 (*n*, %)	26 (7%)
Boolean-based remission	
Boolean 1.0 (*n*, %)	60 (15%)
Boolean 2.0 (*n*, %)	103 (26.2%)
PGA near remission (*n*, %)	111 (28%)
BooleanX (*n*, %)	171 (43.5%)

*Boolean 1.0* TJC ≤ 1, SJC ≤ 1, CRP (mg/dL) ≤ 1, PGA ≤ 1, *Boolean 2.0* TJC ≤ 1, SJC ≤ 1, CRP (mg/dL) ≤ 1, PGA ≤ 2, *BooleanX* TJC ≤ 1, SJC ≤ 1, CRP (mg/dL) ≤ 1, *PGA* Patient Global Assessment, *SJC* swollen joint count, *TJC* tender joint count

### PGA and disease impact

**Fig. 2:**
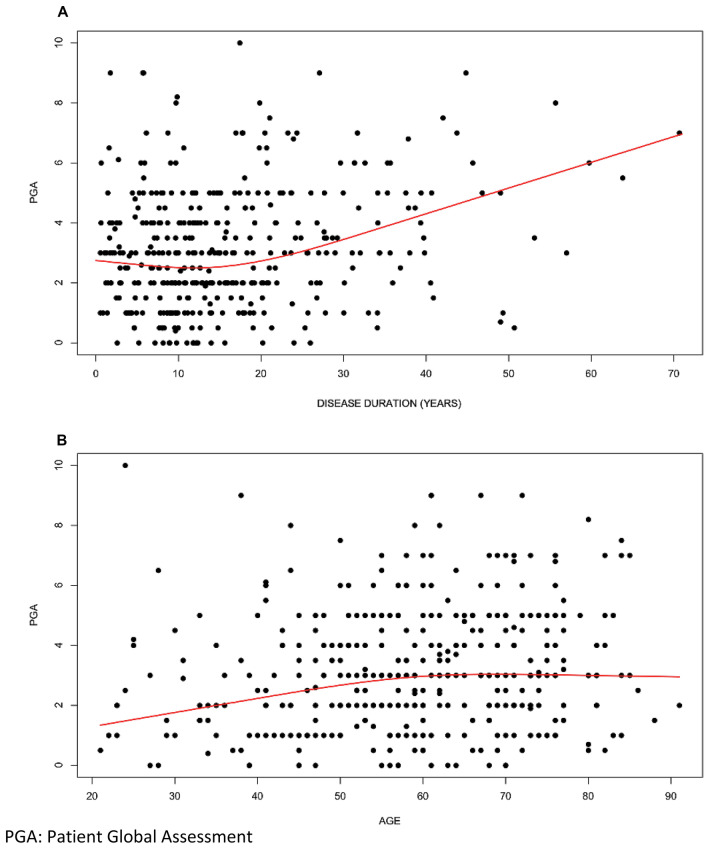
PGA distribution in relation to disease duration (**A**) and age (**B**).PGA: Patient Global Assessment

In univariate analysis as regards disease impact, the analysis displayed a nonlinear relationship between median of PGA and disease duration (contribution of non-linear term *p* = 0.03). A slight decrease of median PGA was observed till about 15 years, starting from a model estimating median 2.75 at duration = 0.55 to 2.54 in about 15 years after diagnosis. Thereafter, an increase of median PGA was observed until 6.88 at 71 years (Fig. 2A). Equal median PGA score was seen in patients with RF and/or ACPA positivity and in patients without RF and ACPA.

The median HAQ score was 0.25 (IQR 0–0.63). At univariate analysis the PGA showed a strong association with the HAQ score (*p* < 0.01): a unitary increase in the HAQ measure was associated with an increase in the median PGA score of 2.67 points (95% C.I. 2.00, 3.25).

The median RAID final value reported in the whole cohort was 2.36 (IQR 0.63–4.54) and it resulted associated with the median PGA score (*p* < 0.01); in detail, the median PGA increase of 0.60 (0.49, 0.71) points for each unitary increase in RAID scale.

The analysis also considered the role of comorbidities in defining PGA. Interestingly, no statistically significant change was observed in the median PGA score in patients with fibromyalgia and depressive symptoms with respect to who do not have such disease (median PGA difference 1 (95% CI. 0.2, 2.5) *p* = 0.081 for fibromyalgia and 0.7 (I.C. 95% − 0.5, 1.5) *p* = 0.155 for depression.

Subjecting the above-mentioned variables to multivariate analyses regression, the final model obtained by backward selection procedure, retained only HAQ and RAID scores which were found as factors associated with the median PGA score; in particular, the HAQ measure displayed a stronger impact on the median PGA (*p* = 0.02, value 0.83; 95% CI 0.45, 1.73). See Table 4.

**Table 4:** Significant results from the quantile regression analyses of the three macro-areas

Disease severity/impact	Value (95% CI)	*P* value
HAQ score	0.83 (0.45, 1.73)	0.02
RAID total	0.44 (0.32, 0.53)	< 0.01
Disease activity		
Low-dose GC	0.43 (0.00, 0.75)	0.02
RAID_pain	0.34 (0.25,0.43)	< 0.01
RAID_sleep	0.22 (0.14, 0.32)	< 0.01
Socio-demographic factors/lifestyle		
RAID physical well-being	0.6 (0.38, 0.70)	< 0.01

*HAQ* health assessment questionnaire, low-dose GC (glucocorticoids): ≤ 7.5 mg/day prednisone or equivalent, *RAID* rheumatoid arthritis impact of disease score

### PGA and disease activity

Swollen joint count (SJC) and tender joint count (TJC) at clinical evaluation are related to PGA scores. The univariate analysis highlighted that the median PGA score in subjects with SJC ≥ 1 was significantly different (*p* < 0.01) from those with normal physical examination with an increase of about 1.2 (95% CI 0,2) points. Similarly, also the median PGA score in subjects with TJC ≥ 1 resulted significantly different (*p* < 0.01) from patients without tender joints, with an increase of about 2 (95% CI 1.0, 2.5) points.

As regards ongoing medication, the use of low-dose glucocorticoids and NSAIDs was found to be significantly associated with PGA (respectively *p* < 0.01 and *p* = 0.03). Specifically, the use of NSAIDs increased the median PGA score of 1 (95% CI 0.5, 2.3) unit in comparison with NSAIDs non-users, while ongoing therapy with low-dose glucocorticoids increased the median PGA score of 1.5 (95% CI 0.5,2) units compared to the subjects who did not need steroids.

Pain NRS value was one of the strongest drivers of the RAID scale in the definition of PGA, with a median increase of 0.54 (95% CI 0.45, 0.67) in PGA score for a unitary increase of pain NRS value (*p* < 0.01). Similarly, patients who reported higher levels of sleep difficulties displayed an increased median PGA score (*p* < 0.01, value 0.5 (95% CI 0.33, 0.58)).

The final model of multivariate regression analysis showed that, considering the effect of the diverse variables, the use of low-dose corticosteroids and the scores of pain and sleep difficulties items assessed by the RAID questionnaire were associated with median PGA.

### PGA and socio-demographic factors/lifestyle

The median PGA showed a peculiar non-linear relationship with age at the time of the assessment. The contribution of the non-linear term was statistically significant (p = 0.046).

People aged 21–55 had a median PGA that tended to increase (from 1.37 at 21 years to 2.85 at about 55 years), while after about 55 years of ageit remained stable around 3 (Fig. 2B). The median PGA score was equal in patients classified upon sex and BMI (3 for all categories). For education the difference between university and lower education level was − 0.5 (95% CI 1,0, *p* = 0.21).

At univariate analysis, physical activity was found to be associated with the median PGA (*p* = 0.002). In particular, for the duration of the physical activity the coefficient is − 1 (95% CI − 1.1,0). The subgroup of patients who reported regular physical activity (> 2 times/week) displayed a median PGA decreased by approximately 1 point when compared with those with a sedentary lifestyle and those who reported 1 or 2 times of weekly exercise.

Consistently, patients reporting a worse score of RAID physical well-being had higher median PGA (*p* < 0.01) (coefficient value: 0.55 (95% CI 0.44,0.67)). Nevertheless, only RAID physical well-being resulted independently associated with median PGA (*p* < 0.01) in multivariate analyses regression. Significant results from the multivariate regression analyses of each macro-area are shown in Table 4.

## Discussion

Our results support the relevant contribution of PGA in defining disease remission by using the overall Boolean-based remission rate (1.0 version 15% vs 2.0 version 26.2%). These data are concordant with previous literature with a better agreement with the SDAI definition (28%); this rate was even higher considering also near remission state, roughly 43.5% [12, 16, 31, 32].

Moreover, we confirmed that the level of agreement between patients and physicians rating disease activity is not optimal, in line with the published literature regarding patient–physician discordance [33–36].

The importance of incorporating the patient's perspective into the disease activity criteria is out of dispute. Notwithstanding, it’s of paramount importance to be aware of potential contextual factors that should be recognized to interpret properly the patient's rating [3, 7].

Among factors related to RA disease activity, the intake of low doses of GCs was significantly related to PGA score, contrary to the use of NSAIDs, often administered for degenerative musculoskeletal complications. Thus, evincing that PGA reflects a non-optimal control of disease with ongoing therapy. In our cohort, physician evaluation of tender and swollen joints did not maintain significance at multivariate analyses, showing to be a lesser predictor of PGA in line with the literature [9, 17].

Furthermore, our results highlighted a strong correlation between RAID total score and PGA [37–39]. Indeed, we found that items “sleep”, “pain” and “physical well-being” correlated with PGA, suggesting that, when asked to evaluate their disease, patients are prone to focus on these aspects. A fair amount of literature underlined that pain is the major cause of distress for RA patients and therefore a pivotal predictor of PGA, reflecting either inflammatory status or structural joint damages [40–43]. As for sleep, it has already been shown that up to 70% of RA patients complain about insomnia or a decreased quality of sleep due to disease activity or the use of medications such as corticosteroids [44, 45]. This is consistent considering that sleep disturbances and pain appear to be linked in a cyclical pattern: indeed, disease activity, increased pain, fatigue and psychological distress might negatively affect daily-life activities leading to sleep disorders, which in turn are hypothesized to contribute to pain sensitivity, mood symptoms and functional impairments creating a cascade of dysfunction [46].

In our study, depressive symptoms did not result predictive for higher PGA scores, as also reported by previous findings [31]. This might be related to the low accuracy of medical records in reporting the prevalence and severity of mental issues in patients with rheumatic diseases. Finally, physical well-being is a multifactorial domain which could be influenced by factors such as pain, sleep, functional disability, and social participation, thus it is not surprising its predictive value on PGA. Besides, this suggests that patients are more likely to interpret PGA as a question about their general health rather than disease specific.

As already reported [42, 43], functional disability investigated with HAQ demonstrated to have a strong correlation with disease impact, with a unitary increase in HAQ score associated with an increase in PGA of 0.83. Noteworthy, HAQ is considered a predictor of disability even at baseline [47].

To note, PGA displayed a peculiar trend at different disease duration: from 0 to 15 years after diagnosis, the median PGA showed a decrease, while in longstanding diseases (> 15 years) higher median of PGA was found. This could be explained by assuming an effective remission of inflammatory symptoms due to treatment during the first years of disease, while the later increase could reflect joint damage, secondary osteoarthritis or onset of other comorbidities, as already suggested by previous studies [8, 40, 48].

Looking beyond disease activity and impact, we found no association with sex, BMI, presence of fibromyalgia (probably underdiagnosed as the diagnosis was derived from medical records) and educational levels. Controversial data about age were observed; indeed, our findings showed that people aged 21–55 had a median of PGA that tended to increase, while after 55 years old it remained stable. In contrast with our results, previous studies indicated that global assessment scores showed an age-dependent increase, with elderly patients expressing higher PGA [11, 49].

Some limitations should be considered. First, even though the sample was quite large, the recruitment in only one Centre might partly affect the generalization of our results; second, when dealing with observational studies, we need to consider missing data and confounding factors; third, self-reported information (e.g., hours of physical activity) might not be always accurate. Furthermore, the absence of radiographic data represents a limiting factor in evaluating disease burden, since bone erosions occurs up to 60% of RA patients and leads to functional impairment, assessed in the study indirectly through HAQ [50].

## Conclusion

These results, in line with previous literature, confirm the need for further investigation to identify relevant priorities for patients and instruments, such as patient decision aid, to empower share-decision in clinical practice and identify adequate assessment of disease impact and disease activity. These core-set might guide clinicians in evaluating the increase of immunosuppressive treatments or otherwise implementation of other additional therapies, such as self-management strategies, thus supporting the proposal of a dual targets (biological and impact) approach [3, 12, 33].

## Data Availability

The datasets analysed during the current study are available from the corresponding author on reasonable request
